# Supplementary material for: Curcumin for Inflammation Control in Individuals with Type 2 Diabetes Mellitus and Metabolic Dysfunction-Associated Steatotic Liver Disease: A Randomized Controlled Trial
Source: Nutrients. 2025 Jun 10;17(12):1972. doi: 10.3390/nu17121972 (PMC12196292; doi:10.3390/nu17121972)
Supplement: Supplementary file 1 [file nutrients-17-01972-s001.zip › nutrients-3668183-supplementary.pdf]

Supplementary materials

SUPPLEMENTAL FIGURE S1 CONSORT 2010 checklist for reporting a randomized trial *

CONSORT 2010 checklist of information to include when reporting a randomized trial*

Section/Topic	Item No	Checklist item	Reported on page No
**Title and abstract**			
	1a	Identification as a randomized trial in the title	1
	1b	Structured summary of trial design, methods, results, and conclusions (for specific guidance see CONSORT for abstracts)	1
**Introduction**			
Background and objectives	2a	Scientific background and explanation of rationale	1-2
	2b	Specific objectives or hypotheses	2
**Methods**			
Trial design	3a	Description of trial design (such as parallel, factorial) including allocation ratio	3
	3b	Important changes to methods after trial commencement (such as eligibility criteria), with reasons	NA
Participants	4a	Eligibility criteria for participants	3-4
	4b	Settings and locations where the data were collected	3
Interventions	5	The interventions for each group with sufficient details to allow replication, including how and when they were actually administered	3-4
Outcomes	6a	Completely defined pre−specified primary and secondary outcome measures, including how and when they were assessed	5-6
	6b	Any changes to trial outcomes after the trial commenced, with reasons	NA

Sample size	7a	How sample size was determined	6
	7b	When applicable, explanation of any interim analyses and stopping guidelines	NA
Randomization:			
Sequence generation	8a	Method used to generate the random allocation sequence	6
	8b	Type of randomization; details of any restriction (such as blocking and block size)	6
Allocation concealment mechanism	9	Mechanism used to implement the random allocation sequence (such as sequentially numbered containers), describing any steps taken to conceal the sequence until interventions were assigned	6
Implementation	10	Who generated the random allocation sequence, who enrolled participants, and who assigned participants to interventions	6
Blinding	11a	If done, who was blinded after assignment to interventions (for example, participants, care providers, those assessing outcomes) and how	6
	11b	If relevant, description of the similarity of interventions	NA
Statistical methods	12a	Statistical methods used to compare groups for primary and secondary outcomes	7
	12b	Methods for additional analyses, such as subgroup analyses and adjusted analyses	7
**Results**			
Participant flow (a diagram is strongly recommended)	13a	For each group, the numbers of participants who were randomly assigned, received intended treatment, and were analyzed for the primary outcome	7
	13b	For each group, losses and exclusions after randomization, together with reasons	7
Recruitment	14a	Dates defining the periods of recruitment and follow−up	NA
	14b	Why the trial ended or was stopped	NA
Baseline data	15	A table showing baseline demographic and clinical characteristics for each group	3-4

Numbers analyzed	16	For each group, number of participants (denominator) included in each analysis and whether the analysis was by original assigned groups	7
Outcomes and estimation	17a	For each primary and secondary outcome, results for each group, and the estimated effect size and its precision (such as 95% confidence interval)	8-9
	17b	For binary outcomes, presentation of both absolute and relative effect sizes is recommended	NA
Ancillary analyses	18	Results of any other analyses performed, including subgroup analyses and adjusted analyses, distinguishing pre−specified from exploratory	9
Harms	19	All important harms or unintended effects in each group (for specific guidance see CONSORT for harms)	15
**Discussion**			
Limitations	20	Trial limitations, addressing sources of potential bias, imprecision, and, if relevant, multiplicity of analyses	12-13
Generalizability	21	Generalizability (external validity, applicability) of the trial findings	13
Interpretation	22	Interpretation consistent with results, balancing benefits and harms, and considering other relevant evidence	10-13
**Other information**			
Registration	23	Registration number and name of trial registry	13
Protocol	24	Where the full trial protocol can be accessed, if available	NA
Funding	25	Sources of funding and other support (such as supply of drugs), role of funders	13

Citation: Schulz KF, Altman DG, Moher D, for the CONSORT Group. CONSORT 2010 Statement: updated guidelines for reporting parallel group randomised trials. BMC Medicine. 2010;8:18.

© 2010 Schulz et al. This is an Open Access article distributed under the terms of the Creative Commons Attribution License (http://creativecommons.org/licenses/by/2.0), which permits unrestricted use, distribution, and reproduction in any medium, provided the original work is properly cited.

*We strongly recommend reading this statement in conjunction with the CONSORT 2010 Explanation and Elaboration for important clarifications on all the items. If relevant, we also recommend reading CONSORT extensions for cluster randomised trials, non−inferiority and equivalence trials, non−pharmacological treatments, herbal interventions, and pragmatic trials. Additional extensions are forthcoming: for those and for up−to−date references relevant to this checklist, see www.consort−statement.org.\

SUPPLEMENTAL FIGURE S2 CONSORT flow diagram of participant enrollment and allocation

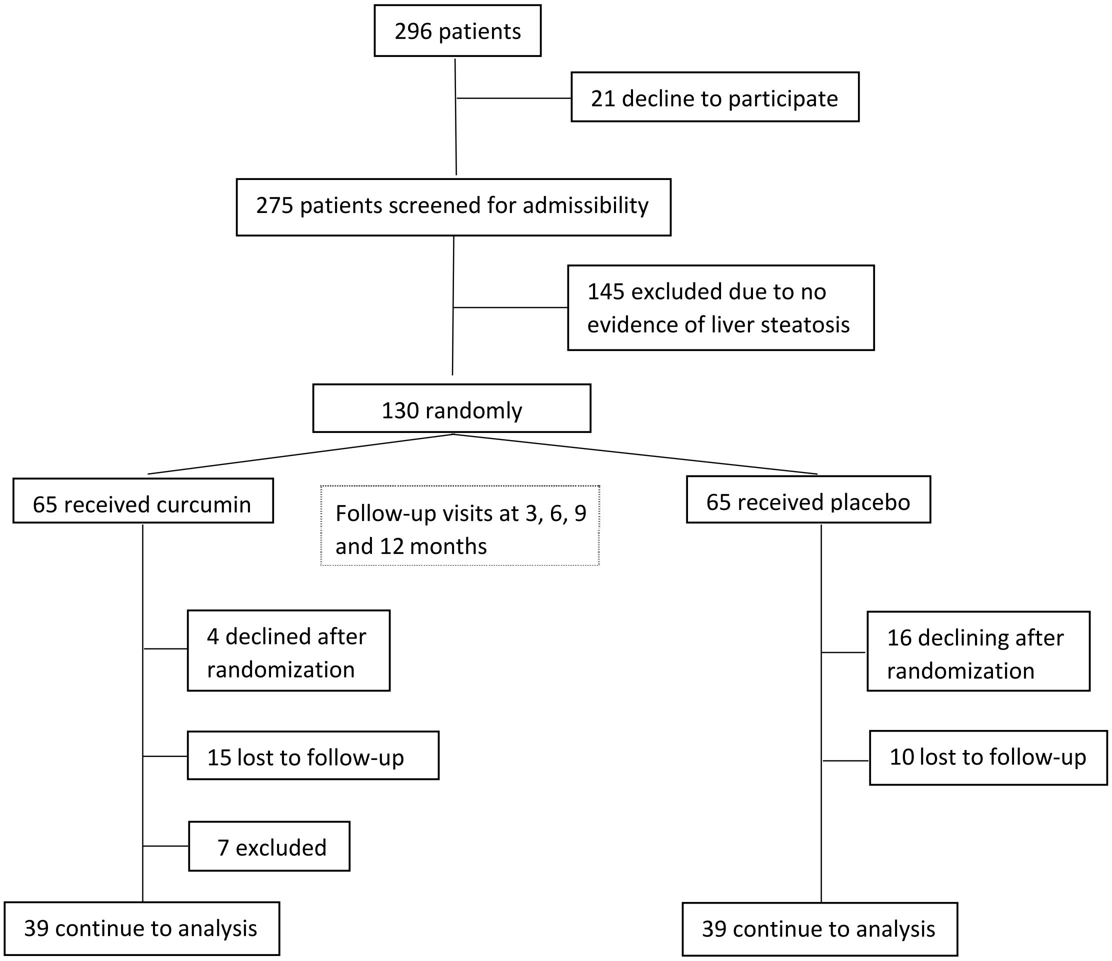

SUPPLEMENTAL FIGURE S3 High−performance thin−layer chromatography profiles of curcuminoid extracts compared with standard curcuminoid chromatograms

The high−performance thin−layer chromatography (HPTLC) chromatogram of the Thai Government Pharmaceutical Organization (GPO) extracts is shown in figure 2, compared with HPTLC chromatogram of standard across curcumin (curcuminoids) in figure 1. In every batch of GPO curcuminoids extract, the peak ratio of curcumin to demethoxycurcumin to bisdemethoxycurcumin was controlled to be 1 to not more than 0.6 to not more than 0.4.

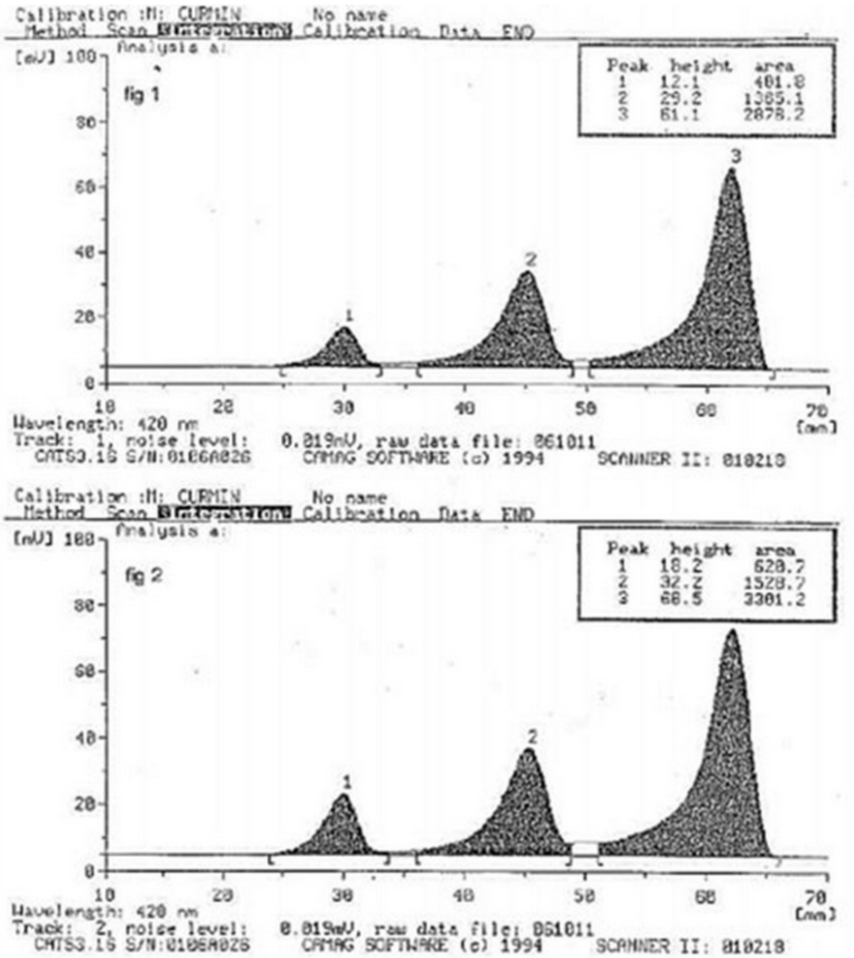

SUPPLEMENTAL TABLE S1 Mean daily nutrient intake of participants at baseline and 12 months by group

Daily intake of nutrients	Placebo (*n*=114)		Curcumin (*n*=113)		*P* value[2]
	Baseline[1]	12 months	Baseline[1]	12 months	
Energy (kcal/d)	1857.60±110.97	1893.74±74.30	1864.21±87.98	1881.16±67.23	0.100
Carbohydrate (% of energy)	57.50±2.57	58.07±2.56	57.04±1.52	58.08±1.62	0.148
Protein (% of energy)	12.98±2.13	13.21±1.35	13.33±1.28	13.44±1.20	0.418
FAT (% of energy)	28.27±2.12	28.91±1.91	28.47±2.42	28.44±2.23	0.056
Fiber (g/d)	8.54±1.16	8.46±0.88	8.49±0.82	8.39±0.64	0.464

[1] All parameters are presented as means ±SDs. There are no significant differences between two groups at baseline for any variable by *t* test.

[2] The curcumin had no significant effect on mean daily intake of nutrients by one−factor ANCOVA with the baseline value as the covariate.

There were no significant differences in the daily mean−energy (energy, carbohydrate, protein, fat, and fiber) and nutrient intakes between the curcumin and placebo groups.

SUPPLEMENTAL TABLE S2. Sex−stratified linear regression analysis of curcumin effect on change in outcomes at 12 months

ΔOutcomes[†]	Males			Females		
	**β***	**95% CI**	***P*−value**	**β***	**95% CI**	***P*−value**
ΔTNF (pg./ml)	−2.76	(−4.92, −0.60)	0.014	−3.90	(−5.57, −2.22)	<0.001
ΔIL1−β (pg/ml)	−0.63	(−0.84, −0.42)	<0.001	−0.73	(−0.93, −0.53)	<0.001
ΔIL−6 (pg/ml)	−7.06	(−9.86, −4.25)	<0.001	−10.2	(−15.70, −4.62)	<0.001
ΔGPx (U/l)	6455	(4540, −8370)	<0.001	7098	(5645−8551)	<0.001
ΔSOD (U/ml)	163	(123,202)	<0.001	137	(112−162)	<0.001
ΔMDA (μmol/l	−1.07	(−1.62, 0.52)	<0.001	−1.02	(−1.47, −0.57)	<0.001
TBF (%)	−3.27	(−5.62, −0.92)	0.007	−3.41	(−5.22, −1.32)	0.001
WC (cm)	−6.15	(−9.04, −3.26)	0.002	−6.21	(−10.8, −1.60)	0.009
NEFA(μmol/l)	−0.46	(−0.72, −0.19)	0.002	−0.38	(−0.66, −0.11)	0.007
BMI (kg/m^2)	−2.58	(−4.46, 0.70)	0.006	−2.97	(−4.74, −1.20)	0.001
Glucose (mg/dl)	−16.4	(−28.35, −3.85)	0.010	−10.3	(−20.4, −0.56)	0.038
HbA1C (%)	−0.95	(−1.75, −0.15)	0.020	−0.46	(−0.83, −0.08)	0.017
Liver stiffness (kPa)	−2.06	(−3.69, −0.44)	0.013	−2.83	(−4.71, 0.95)	0.004
CAP score (dB/m)	−72.5	(−113.0, −32.3)	<0.001	−63.6	(−90.30, −36.80)	<0.001
FLI	−32.8	(−45.4, −20.2)	<0.001	−25.7	(−35.60, −15.8)	<0.001
HSI	−8.70	(−13.1, −4.28)	<0.001	−8.32	(−10.90, −5.74)	<0.001
LAP	−26.1	(−42.66, −9.55)	0.002	−24.1	(−38.45, −9.75)	0.001

[†] ΔOutcomes calculated as the difference between 12−month follow−up and baseline.

***** β (beta) represents the regression coefficient.

All models were adjusted for age.

BMI=body mass index; CAP=controlled attenuated parameter; GPx=glutathione peroxidase; FLI=Fatty liver index; HbA1C=glycated hemoglobin; HSI=Hepatic Steatosis Index; IL−1β = Interleukin−1 beta; IL−6=interleukin−6; IQR Interquartile range; LAP=Lip accumulation product; MDA=malondialdehyde; NEFA=non−esterified fatty acid SOD=superoxide dismutase; TBF= Total Body fat; TNF−α=tumor necrosis factor−alpha; WC = Waist circumference

SUPPLEMENTAL TABLE S3: Accuracy of blinding guesses by participants and onvestigators

Group	Total Respondents	Correct Guesses	Accuracy (%)	*P*–value
Participants	78	38	48.7%	0.84
Investigators	78	41	52.6%	0.73

P–values were calculated using a two–sided exact binomial test, assessing whether the proportion of correct guesses significantly deviated from the expected 50% under the null hypothesis of random guessing.

SUPPLEMENTAL TABLE S4*:* Renal and hepatic adverse effects of curcumin and placebo at each follow–up visit

Variables	Visits	Placebo		Curcumin		*P*–value
		Mean (SEM)	Min–max	Mean (SEM)	Min–max	
Creatinine (mg/dL)	Baseline	0.82 (0.03)	0.42–1.67	0.85 (0.03)	0.45–1.6	0.75
	3 months	0.81 (0.02)	0.41–1.78	0.91 (0.02)	0.46–1.76	0.64
	6 months	0.85 (0.02)	0.45–1.89	0.92 (0.02)	0.52–1.70	0.40
	9 months	0.83 (0.03)	0.44–1.86	0.91 (0.02)	0.52–1.81	0.56
	12 months	0.82 (0.02)	0.41–1.72	0.85 (0.02)	0.43–1.68	0.78
AST (U/L)	Baseline	25.31 (0.82)	11–89	25.34 (0.80)	13–67	0.58
	3 months	22.39 (0.81)	9–78	23.82 (0.88)	11–92	0.07
	6 months	23.42 (0.92)	8–79	24.18 (0.98)	10–89	0.43
	9 months	23.69 (0.84)	13–76	24.28 (0.89)	12–87	0.76
	12 months	23.01 (0.87)	11–89	24.42(0.81)	13–67	0.62
ALT (U/L)	Baseline	26.78 (1.26)	5–121	28.09 (1.48)	6–119	0.09
	3 months	25.08 (1.19)	6–114	26.49 (1.7)	6–132	0.13
	6 months	25.34 (1.15)	7–110	26. 84 (1.64)	7–132	0.21
	9 months	25.67 (1.18)	6–117	27.31 (1.80)	6–127	0.34
	12 months	26.38 (1.21)	5–145	29.14 (1.67)	6–123	0.29

ALT=alanine transaminase; AST=aspartate aminotransferase

SUPPLEMENTAL TABLE S5 Capsule consumption per 3–month interval and daily intake at follow–up visits

Consumption	Visit	Placebo		Curcumin		*P* value
		**Number of subjects who showed up**	**Number of capsules taken (mean, SEM)**	**Number of subjects who showed up**	**Number of capsules taken (mean, SEM)**	
Consumption per 3 months	3–month visit	39	524.7 (54.59)	39	527.4 (52.11)	0.48
	6–month visit	39	521.1 (21.61)	39	526.5 (25.61)	0.15
	9–month visit	39	513.9 (20.32)	39	514.8 (21.33)	0.39
	12–month visit	39	525.6 (20.82)	39	528.3 (21.78)	0.35
Consumption per day	3–month visit	39	5.83 (0.60)	39	5.86 (0.58)	0.62
	6–month visit	39	5.79 (0.23)	39	5.85 (0.28)	0.15
	9–month visit	39	5.71 (0.22)	39	5.72 (0.24)	0.56
	12–month visit	39	5.84 (0.22)	39	5.87 (0.24)	0.54
